# 

**Published:** 1871-07

**Authors:** 


### BOOK NOTICES.

"Paralysis and other Nervous Diseases," $1, by Dr. GEORGE H. TAYLOR, New York. This book is heartily commended to all who have lost the use of their limbs, in whole or in part; it shows the kinds of ailments which can be benefited or cured. The style of the book is unfortunate for common readers; it is not clear and plain enough; but Dr. TAYLOR is a good and honest physician; he will mislead no one by false promises: he is a man of great skill, is enthusiastic in his profession, and has been eminently successful. He has the confidence of all who know him.

"London Lancet," $5 a year, republished monthly by WM. C. HERALD, 52 John St., New York. No. 5, for 1871, contains a rich variety of original and selected articles: Rutherford's Lectures on Experimental Physiology, Irritation, Cause of Scarlatina, Sudden Death from Large Dose of Chloral Hydrate, Action of Light in Small Pox, Vaccination and Re-vaccination, Hospital Reports.

"A Poet's Bazaar," being Pictures of Travel in Germany, Italy, Greece, and the Orient, by HANS CHRISTIAN ANDERSEN, 343 pp., 12mo, $1.75. Published by HURD & HOUGHTON, 13 Astor Place, New York, at Cambridge, Riverside Press. To all who have been abroad, to all who propose going abroad this summer, the perusal of this book will be a source of interest, instruction, and amusement, from title-page to "Finis." It is scarcely possible that any intelligent reader should regret its purchase, for it will enable him to live over the past pleasurably, or prepare him for travel if he is on his first tour.

The office of the Editor is at number forty, Broadway, New York, room forty-eight, from ten to three, daily.

